# The impact of directors’ information technology experience on the financing constraints of internet start-ups—Data mining with python on the China’s New OTC market

**DOI:** 10.1371/journal.pone.0308325

**Published:** 2024-08-09

**Authors:** Huiji Xu, Senqiang Wang, Jianping Wang, Xiuyu Liu, Kaoxun Chi

**Affiliations:** 1 Business School, Shandong Xiehe University, Jinan, China; 2 Business School, Shandong University of Technology, Zibo, China; 3 School of Economics and Management, North China Institute of Science and Technology, Langfang, China; COMSATS University Islamabad - Wah Campus, PAKISTAN

## Abstract

In the era of Internet information technology, the IT background of directors plays a significant role in corporate governance. However, existing research lacks sufficient discussion on the financing constraints faced by Internet startups. This paper examines Internet entrepreneurial enterprises listed on the New Third Board as the research sample. Using content analysis coding methods and Python software for text mining, the study empirically analyzes the impact of directors’ IT backgrounds and media reports on the financing constraints of these enterprises. The results indicate the following: First, directors with IT backgrounds help reduce the financing constraints of Internet startups. The higher the directors’ proficiency in information technology, the more favorable it is for obtaining financing. Second, directors with IT backgrounds have a significant positive impact on the tone of media reports. Third, the tone of media reports reflects the spirit and development of the enterprises, serving as a mediating factor between directors’ IT backgrounds and the financing constraints of the enterprises. The findings of this study are valuable for guiding Internet startups to better leverage the IT expertise of their directors. Additionally, they provide useful insights for reducing the financing constraints faced by these startups.

## 1. Introduction

For startups, successfully obtaining financing is the most important milestone and turning point in their development process. Compared with high-tech enterprises that possess extensive hard technologies, Internet startups are more focused on discovering and serving customer value through business model innovation and have lower investments in hard assets. Consequently, they often face higher financing difficulties, resulting in a high failure rate. Therefore, it is important to understand the factors that could reduce the financial constraints of Internet startups and improve their chances of successful financing.

Regarding the answer to this question, many investment and financing institutions currently believe that the background of the entrepreneurial team members is a more critical factor in deciding whether to invest in a project than the nature of the Internet venture itself. Entrepreneurial teams with strong information technology (IT) backgrounds typically receive more favorable consideration from investors. Theoretical studies also emphasize this point. For instance, Yuan et al. argues that directors with IT backgrounds possess higher decision-making power and can help firms make effective financing decisions [1]. Similarly, Hadi et al. suggest that IT can improve firm performance and attract investors [2]. While both theory and practice recognize that the IT background of the entrepreneurial team positively influences the financing of the entrepreneurial firm, IT background does not necessarily equate to IT competence. If the IT background of the entrepreneurial team is not fully understood or is misinterpreted, it may not be helpful for financing activities. Therefore, an intermediary is needed to better convey the IT competence of the entrepreneurial team to investment and financing institutions.

With the widespread application of new media technology, the influence of media reports on business operations has become increasingly prominent. The impact of media reports on corporate finance, in particular, has garnered significant scholarly attention. For example, Martin and Flores argue that positive media reports and external governance pressure can positively influence and improve the financing structure of enterprises [3]. Similarly, Yang et al. found that optimistic media reports convey positive emotions to investors, thereby motivating their investment behavior [4]. Given the importance of media coverage, several scholars have analyzed the proactive management behavior of corporate management towards media coverage. Shao and Cai argue that firms’ strategic management of media information can boost share prices and increase the probability of successful mergers and acquisitions (M&A) and restructuring [5]. Additionally, Stewart notes significant media disclosure management behaviors by firms, with differences in these behaviors across industries [6]. High-tech firms, in particular, exhibit more prominent media disclosure management tendencies.

In summary, it is clear that media coverage can play a very effective connecting role in the relationship between entrepreneurial teams’ information technology experience and entrepreneurial firms’ financing. However, current research has mostly overlooked the influence of the entrepreneurial team’s information technology experience on corporate finance constraints, and the mediating role of media reports has also been less explored. Accordingly, this study takes the New Third Board Internet startups as the research object to explore the impact of directors’ information technology experience on corporate finance constraints by considering the mediating role of media reports in this nexus. Exploring this association will not only help to enrich the theoretical research framework on the relationship between the information technology experience and financial constraints but also provide insights to better carry out the financing activities of Internet startups.

## 2. Theoretical analysis and research hypotheses

### 2.1 Relationship between directors’ information technology experience and media coverage

With the development of science and technology, the media has become increasingly influential in economic and social life, and has become an important way for people to know the world. For business operations, media coverage is a powerful weapon used to show that it meets the expectations of stakeholders, and is seen as an effective way for companies to develop and maintain friendly relations with external investors or stakeholders. Media coverage is often used by business executives to alleviate the asymmetric information environment between the company and the outside world. Internet startups in particular value this and see media coverage as an important channel to communicate their development and prospects to the outside world. Internet startups are characterized by higher operational uncertainty, but when the founders or their board members have a rich background in information technology, it often means that the firms have more reliable growth potential, and thus they receive more positive media coverage.

For example, Hao et al. highlight that independent directors with an information technology(IT) background can integrate internal and external resources [7], providing professional advice and suggestions for corporate innovation, and exert the "expert effect" to enhance corporate innovation performance. This, in turn, can lead to positive media coverage. Similarly, Bendig et al. found that IT can help increase corporate sales and profitability [8], which also attracts favorable media coverage. On the other hand, in the process of enterprise development, annual report indicators and other public information have a very significant role in guiding media coverage. In addition, the board of directors, as the highest decision-making and controlling body of the enterprise, which has the obligation and incentive to monitor the enterprise’s media coverage and respond to media tendencies in a timely manner plays a significant role in the media coverage. When the board of directors of Internet startups has a rich background in information technology, board members can be frequently exposed to the Internet and familiar with Internet communication, and thus they are expected to be more responsive to media coverage. In conjunction with Bygstad and Aanby’s study [9], the use of information technology by directors can effectively integrate various types of data and information within corporate activities, enhancing the timeliness and effectiveness of corporate communication. This improved communication is conducive to attracting positive media coverage. Thus, this study proposes the following hypotheses on the relationship between directors’ information technology experience and media coverage:

H1: The richer the director’s background in information technology, the more positively the media will report on Internet startups.

### 2.2 The relationship between directors’ information technology experience and financing constraints of Internet start-ups

Financing constraints refer to the degree of restriction faced by enterprises in carrying out external financing. High financing constraints mean that it is more difficult for enterprises to raise funds from the outside world, and the process of financing will increase the cost of financing, which will easily make enterprises fall into a financial crisis. Low financing constraints indicate a favorable financing environment, which will bring sufficient financial support to the enterprise.

The richer the information technology experience of the directors, the more conducive it is for Internet start-ups to think strategically about the information environment. Directors with information technology experiences not only understand information technology knowledge but also tend to be very good at the overall operation of the enterprise and strategic planning. Hence, they will establish an advantage of information technology resources within the enterprise from the perspective of the long-term development of the enterprise and the maximization of interests, and promote financing activities based on the advantage of this resource. Mojambo et al. indicate that executives with an information technology (IT) background have a better understanding of the firm’s production processes and technological advantages [10]. They focus on improving the firm’s productivity, which facilitates financing or refinancing. Boards of directors with IT backgrounds guide the firm’s development path through strategic planning, thereby favoring the achievement of corporate financing objectives.

Regarding information disclosure, directors with IT backgrounds positively influence the decision to proactively disclose social responsibility reports. This helps strengthen communication between enterprises and the external environment, reducing the information asymmetry faced by investors. Zhang et al. found that executives with IT backgrounds can alleviate information asymmetry, lower the cost of resource acquisition, and ease corporate financing constraints [11]. Similarly, Zhou et al. noted that directors with IT expertise are more likely to select promising innovation projects and effectively supervise the innovation process, thus facing fewer financing constraints [12].

The characteristics of IT backgrounds among board members enhance the board’s control and monitoring of various aspects of the firm’s information, all of which are beneficial to financing activities. For example, Héroux argues that directors with IT backgrounds can mitigate information risk, promote firm innovation, and assist in raising finance [13]. Therefore, when directors have a rich IT background, they are more likely to understand the operation of information technology and information systems, pay more attention to the status of various information reports of the firm, and seek more external support for the firms to meet their financing needs.

As a result, this study proposes the following hypotheses about the relationship between directors’ information technology background and financing constraints of Internet start-ups:

H2: The richer the information technology experience of directors, the lower the financing constraints faced by Internet startups.

### 2.3 The mediating role of media coverage on the relationship between directors’ information technology experience and financing constraints of Internet startups

Media reports, as the facade of corporate development, influence the psychological construction of investors’ investment behavior. Reports about the development of a thriving enterprise will subconsciously drive investors to invest profitably; while reports about the development of a precarious enterprise may hinder the investments in that enterprise. In the current information dissemination speed, a wide range of circumstances, some of the public, real information about the enterprise report by reproducing, invariably for investors to build up the information of the "mimicry environment". Lipman, the author of "mimetic environment", believes that the information environment formed by mass communication activities is not a mirror-like reproduction of the objective environment, but an environment provided to people after the mass media select, process, and report news and information and re-structure them.

With the power of the media, the information about the prosperous development of enterprises is timely disseminated to the general public, and the information about the business difficulties and countermeasures of enterprises is explained to the public in a reasonable and justified manner, which can bring positive affirmation from the public for the business activities of enterprises. This can vigorously promote the development of enterprise financing activities. For example, Yu et al. find that non-negative media reports can enhance the quality of internal control, thereby facilitating business credit financing [14]. Fu et al. demonstrate the significant impact of media on public perceptions and societal attitudes, influencing firms’ valuation expectations and investor behavior [15]. Additionally, media reports enhance the financing efficiency of Internet startups through two facilitation mechanisms: first, by objectively disclosing listed firms’ operations, thereby providing insights into their internal workings; second, through the media’s external monitoring of firms’ operations. These mechanisms provide investors with valuable reference and judgment information on enterprise operations, reducing investment risk due to information asymmetry, and influencing investor sentiment and behavioral decisions, thus alleviating financing constraints. Yan et al. further note that listed companies can effectively utilize media to release pricing information at optimal times, thereby enhancing market pricing efficiency [16].

In related theoretical studies, Dyreng et al. found that there is active management of media disclosure during corporate executives’ shareholding reductions, allowing corporate media coverage to rise abnormally in the short term, which in turn drives up the corporate share price in order to realise the goal of wealth transfer [17], while Yu argues that financial PR firms will manage media information for the firm during IPOs, with the aim of obtaining a higher level of media attention and a more positive reporting bias [18]. Therefore, the tendency of positive media reports is conducive to publicizing more positive situations of corporate operations to investors, thus getting more affirmation from investors, creating a positive investment expectation for the future development of the enterprise, and will be more willing to invest for the enterprise.

Further synthesizing the derivation of the previous hypotheses, this study suggests that media coverage plays a mediating role in the relationship between directors’ information technology experience and financing constraints, i.e., directors with information technology experiences utilize their information technology strengths to prioritize the observation of affective tendencies in media coverage, and then make timely adjustments to attract outside investment. As a result, this study proposes the following hypotheses:

H3: Media coverage mediates the relationship between directors’ information technology experience and financing constraints.

## 3. Research design

### 3.1 Sample selection and data sources

This study selects Internet entrepreneurial enterprises listed on the New Third Board as its research sample. Data collection is conducted through coding work followed by empirical research. The specific sample selection process is detailed as follows: Firstly, defining startup enterprises draws on Yang et al., where enterprises with a time interval between establishment and listing date of up to five years (≤5 years) are considered startups [19]. Secondly, defining the Internet industry refers to the National Bureau of Statistics of China’s economic industry code, specifically identifying enterprises under code I64 as Internet enterprises. Thirdly, to ensure comprehensive media coverage over a full year, the study includes enterprises listed between 2012 and 2019. Applying these criteria, the study initially identifies 105 Internet startups as the original sample, with further refinement to eliminate incomplete data, resulting in a final empirical analysis sample of 102 NSSB Internet startups.

In this study, the coding criteria for educational and practitioner backgrounds are defined as follows: On the one hand, the study defines an information technology background based on undergraduate majors listed in the 2021 Catalogue of Undergraduate Majors of Ordinary Higher Education Institutions released by the Ministry of Education. Specifically, majors included are professional electronic information (0807), computer (0809), management science and engineering (1201), information management and information system (120102), and e-commerce (1208). On the other hand, this includes work experience in various fields related to IT and information technology. Specifically, it encompasses roles in IT information technology, information management, information system, information technology construction, ERP construction, software development, internet and network development, computer engineering, electronic engineering, system engineering, system construction, e-commerce, e-government, and Internet of Things cloud computing. Once these definitions were established, the study formed a coding team to conduct double-blind coding to assess the backgrounds of directors from the sample of 102 firms.

Regarding the definition of affective tendencies of media reports, this study uses crawler software to crawl relevant media reports of the sampled firms. The time interval of the media reports was set as the reports in a complete financial year after the publication date of the public prospectus. On this basis, Python and CM6 data cleaning and JIEBA segmentation tool were used to analyze the sentiment of the obtained text and calculate the sentiment value. Finally, all the reported sentiment values of the sample cases were summed up to get the final sentiment value of the sample cases, with positive sentiment as positive and negative sentiment as negative.

### 3.2 Definition of variables

#### 3.2.1 Dependent variable: Financing constraints (SA)

Financing constraints are difficult to quantify accurately, and representative of previous studies are the investment-cash flow model, the cash-cash flow model, the KZ model, and the current newly developed WW index and SA index. Among them, the SA index utilizes two elements of firm size and firm age to model financing constraints, which is more in line with investors’ measurements of firms and also helps to reduce the endogenous problem of the model. In view of this, this study uses the SA index to measure financing constraints. The SA index is calculated as (Eq 1):

$$SA=0.043\times {\left(\mathrm{size}\right)}^{2}-0.040\times \mathrm{age}-0.037\times \mathrm{size}$$ (1)

Size here refers to the natural logarithm of firm size. Measures of firm size include sales, number of employees, total assets, net worth, market value of stocks and bonds, cost of goods sold, number of sub-firms, and value added by the firm, of which the main three are sales, number of employees, and total assets. Since sales are neutral to the input ratio of factors of production and reflect the fluctuation of demand in a certain period of time. Therefore, this study uses sales as a measure of firm size. Sales are selected for a complete financial year after the year of issue of the public prospectus. Here age denotes the age of the firm. The SA index is negative, with smaller values indicating a greater degree of financing constraints and larger values indicating a lesser degree of financing constraints.

#### 3.2.2 Intermediate variable: Media coverage (REV)

As the data collected in the important national newspaper media in the previous period were sparse and mostly showed zero data vacancy, thus this study collects the media reports of the sample cases through Internet resources, which mainly focus on Baidu financial news information. For the specific measure, this study draws on Ettredge et al.’s approach [20], which uses the sentiment value of media coverage as a quantitative indicator, expressed as REV.

#### 3.2.3 Dependent variable: Directors’ information technology experience value (DTB)

In conjunction with the analysis above, this study utilized an organizational coding group to code and collate the number of directors with IT backgrounds on the boards of 102 listed firms. This data was then quantified using a percentage calculation, reflecting the proportion of directors with IT backgrounds relative to the total number of board members in each firm.

#### 3.2.4 Control variables

With reference to previous studies, the following variables were selected as control variables in this study. These include time of establishment (in years) (FT), return on equity (ROE), gearing ratio (DAR) and registered capital (RC).

The definitions and computational descriptions of the variables in this study are shown in Table 1.

**Table 1:** Definitions of key variables and description of measurement methods.

Variant	Variable name	Variant	Description of calculations
Implicit Variable	SA index	SA	Drawing on Sun’s study(2019), please see the text for the equation
Independent Variable	Director’s information technology experience	DTB	Ratio of directors with IT background to total number of board members
	Directors’ information technology experience Classification	DTC	Directors with information technology experience (denoted by "1") Directors without information technology experience (denoted by "0")
Intermediary Variant	Media coverage of sentimental value	REV	Media coverage in the year prior to the public offering computes the word vector, the sum of the positive and negative sentiment values
Containment Variant	Year of establishment	FT	Time from limited liability company formation to listing in years
	return on net assets	ROE	Ratio of net profit to total assets for the year preceding the initial public offering of the enterprise
	gearing	DAR	Ratio of corporate liabilities to total assets
	registered capital	RC	Registered capital in the public transfer prospectus of the enterprise
Clusters Variant	Grouping of independent directors	IDG	Whether the percentage of independent directors is higher than its sample median, in two groups

### 3.3 Modeling

1. For the relationship between directors’ information technology experience and media coverage, this study constructed the following Model 1 to test hypothesis H1:
REV=a+b×DTB+c×Controls+ε Model 1
2. The relationship between directors’ information technology experience and financing constraints. This study constructed the following Model 2 to test hypothesis H2:
SA=a+b×DTB+c×Controls+ε Model 2
3. For the test that media coverage mediates the relationship between directors’ information technology experience and financing constraints, the study constructed the following Model 3 to test hypothesis H3:
SA=a+b×DTB+c×REV+d×Controls+ε Model 3

This paper tests the significance level of the regression coefficients in the causal chain as follows: firstly, Model 2 is constructed to test the impact of directors’ information technology background on financing constraints, and if the regression coefficient is significant, it indicates that directors’ information technology background significantly affects the financing constraints of the enterprise; secondly, Model 3 is constructed to test the impact of the intermediary variable, media reports, on financing constraints, and if the regression coefficient is significant, it indicates that If the regression coefficient is significant, it means that the emotional tendency of media reports has a significant impact on financing constraints; finally, combine the test results of Model 2 and Model 3 to analyse the changes of regression coefficients, if the regression coefficients of directors’ information technology background in Model 2 and Model 3 are significant, and the significance of regression coefficients of directors’ information technology background in Model 3 decreases or even is not significant in comparison with that of directors’ information technology background in Model 2, it implies that Media coverage plays a partially mediating role or even a fully mediating role in the impact of directors’ information technology background on financing constraints, which also implies that the positive guidance of directors with information technology background on the tendency of media coverage will be favourable to the financing status of the enterprise.

## 4. Empirical testing and analysis of results

### 4.1 Descriptive statistics of main variables

The statistics show that: first, among the 102 NSS Internet startups, 76 companies have directors with information technology experience and 26 companies have directors without information technology experience; second, the media coverage of the companies with directors with information technology experience and those with directors without information technology experience have positive sentiment tendency.

### 4.2 Empirical analysis of the impact of directors’ information technology experience on media coverage

#### 4.2.1 T-Value test

Differences in media coverage were comparatively analyzed based on whether any directors in the sample firms had an information technology background. Specifically, the mean value of reported sentiment for enterprises with directors who have an IT background is 1.7855, which is significantly higher than the mean value of 0.3989 for enterprises without such directors. Both mean values are significant at the 1% level, implying that the directors’ IT backgrounds have a significant positive impact on media coverage.

#### 4.2.2 Further tests of the impact of directors’ information technology experience on media coverage

To further test Hypothesis H1, we conducted an empirical analysis using Model 1 above, and the results are shown in Table 2. The regression results show that, controlling for other relevant variables, directors with information technology experience (DTB) have a significant positive impact on the affective tendency of corporate media coverage (REV). This shows that directors with information technology experience help firms to obtain positive media coverage results and hypothesis H1 is valid.

**Table 2:** Influence of directors’ IT background on media coverage.

Variant	REV	
	M1	M2
FT	0.269 (1.110)	0.223 (0.985)
ROE	0.009 (1.595)	0.008 (1.592)
DAR	0.000 (-0.328)	0.000 (-0.159)
RC	0.472*** (4.181)	0.443*** (4.196)
DTB		2.493*** (3.940)
Constant	-0.387 (-0.461)	-0.805 (-1.018)
adj R^2^	0.147	0.258

Note: *, **, *** indicate significant at the 10%, 5% and 1% levels, respectively.

### 4.3 Impact of directors’ information technology experience on financing constraints

The previous empirical tests established that directors’ information technology experience has a significant positive effect on affective tendencies towards media coverage. This study further addresses the hypothesis H2 proposed above by conducting the following tests.

The regression analysis was conducted using Model 2 proposed above to test the effect of directors’ information technology experience on corporate finance constraints while controlling for other relevant factors. The results of the regression analysis are shown in Table 3, and it can be found that the adjusted R^2^ is greater than 0.3, which indicates that the fit of the model is better, and the coefficient of director’s information technology experience (DTB) is positive and significant at the 1% level, which indicates that the higher the value of the director’s information technology experience (DTB) is, the higher the SA index is, and the smaller the degree of the enterprise financing constraints is. That is, directors with information technology experience pay more attention to the financing problem of the enterprise, which is more favorable to the enterprise to obtain financing successfully. Hypothesis H2 is verified.

**Table 3:** Impact of directors’ IT background on financing constraints.

Variant	SA	
	M3	M4
FT	0.308 (1.486)	0.266 (1.390)
ROE	0.016*** (3.315)	0.015*** (3.467)
DAR	0.000 (-0.261)	-0.000 (-0.075)
RC	0.397*** (4.121)	0.370*** (4.162)
DTB		2.276*** (4.263)
Constant	-1.457** (—2.031)	-1.838*** (-2.756)
adj R^2^	0.203	0.323

Note: *, **, *** indicate significant at the 10%, 5% and 1% levels, respectively.

### 4.4 The mediating effect of media coverage on the relationship between directors’ information technology experience and financing constraints

On the basis of the above analysis, this study further excludes the confounding interference of year of establishment, return on net assets, gearing ratio and registered capital, and empirically analyzes the mediating effect of media reports on the relationship between directors’ information technology experience and financing constraints by adopting Model 3 constructed above. The regression results are shown in Table 4: on the one hand, the model-adjusted R^2^ is 0.434, which is a good fit; on the other hand, the coefficient of media coverage’s influence on financing constraints is 0.351 and significant at the 1% level. On this basis, the changes in the significance level of the regression coefficients in the causal chain are further tested according to the test of mediating variables. From the results of Table 4 and Table 4, it can be seen that the director’s information technology experience (DTB) presents significance on financing constraints in both cases, but according to Table 4, it can be seen that the regression coefficient of the director’s information technology experience on the impact of financing constraints under the influence of the mediator variable, the sentiment value of the media reports (REV), decreases from 2.276 to 1.401 and the level of significance decreases, which indicates that the mediator variable in this case The sentiment value of media coverage plays a partial mediating role. It can be seen that directors with an information technology background, due to their familiarity with information and networks, can help the enterprise create a favorable media information environment. This positively influences the market image of the enterprise, enabling it to better attract external investment during the financing period and reduce financing constraints. Therefore, hypothesis H3 is verified.

**Table 4:** The role of media coverage in mediating financing constraints.

Variant	SA	
	M5	M6
FT	0.266 (1.390)	0.188 (1.067)
ROE	0.015*** (3.467)	0.012*** (3.029)
DAR	-0.000 (-0.075)	-0.000 (-0.009)
RC	0.370*** (4.162)	0.215** (2.431)
REV		0.351*** (4.456)
DTB	2.276*** (4.263)	1.401*** (2.664)
Constant	-1.838*** (-2.756)	-1.555** (-2.537)
adj R^2^	0.323	0.434

Note: *, **, *** indicate significant at the 10%, 5% and 1% levels, respectively.

### 4.5 Robustness tests

#### 4.5.1 Robustness tests after removal of special samples

To mitigate the effects of endogeneity, this study adopts the method of removing special firms for robustness testing. Generally, Internet startups with R&D modes are more likely to have board members with information technology backgrounds due to the need for specialized expertise. These firms are considered an extreme sample in this empirical test. Therefore, this study excludes firms with R&D modes from the sample, retaining 70 firms, and re-analyzes these 70 samples according to the previously developed regression model. The results, shown in Table 5, indicate that the model fit is greater than 0.3, consistent with the confidence interval. Furthermore, the directors’ information technology background (DTB) and media coverage sentiment value (REV) continue to have a significant positive effect on the SA value, thereby reducing the financing constraints of the firms. Thus, the impact relationship between directors’ IT backgrounds, media coverage, and financing constraints remains significant, even after accounting for the effects of special industries. This suggests that after excluding Internet firms with an R&D model, directors with information technology experiences still positively affect the results on media coverage and help firms obtain positive media coverage; it also suggests that in firms without an Internet R&D model, directors with information technology experiences can still help firms to absorb external investment. Hypotheses H1, H2, and H3 remain confirmed.

**Table 5:** Impact of directors’ IT background, media coverage on financing constraints (deleting special industries).

Variant	SA	
	M7	M8
FT	0.153 (0.592)	0.052 (0.234)
ROE	0.012** (2.298)	0.009** (2.081)
DAR	0.000 (0.167)	0.000 (0.385)
RC	0.466*** (4.412)	0.244** (2.400)
REV		0.000*** -4.747
DTB		1.539** (2.260)
Constant	-0.999 (-1.118)	-1.047 (-1.376)
adj R^2^	0.244	0.457

Note: *, **, *** indicate significant at the 10%, 5% and 1% levels, respectively.

#### 4.5.2 Robustness test after considering subgroups of independent directors

Independent directors with extensive social resources are, to a certain extent, independent of the interests of all parties in listed companies and can effectively monitor the operators. Mishra argues that a higher proportion of independent directors increases their influence or "voice," enhancing their monitoring role [21]. Consequently, the higher the proportion of independent directors, the higher the quality of accounting information disclosure.

In order to avoid being influenced by independent directors, this study further investigates the heterogeneity of the impact of media coverage on corporate finance constraints of listed Internet startups under different independent director share conditions. First, the judgment criteria of independent directors are based on two characteristics of independent directors: first, independence, which means that the independent directors themselves and their relatives are not related to the company, are not employees or former employees of the company, are not important customers or suppliers of the company, as well as do not have financial or interpersonal relationships with the management of the company; and second, they are required to have good qualities and are free from any illegal or criminal malpractices. Second, according to whether the percentage of independent directors is higher than its sample median, the 102 samples are divided into the group with high percentage of independent directors and the group with low percentage of independent directors, after which the group test is conducted. According to the two groups shown in Table 6, under the influence of independent directors, directors with information technology experience can still help enterprises to create a good media information environment, which helps to show a good market image of the enterprise, which in turn helps the enterprise to better attract external investment during the financing period. Hypotheses H1, H2 and H3 are still confirmed.

**Table 6:** Impact of media coverage on financing constraints after independent directors grouping.

Variant	SA (IDG low)		SA (IDG high)	
	M9	M10	M11	M12
FT	0.089 (0.382)	0.039 (0.184)	0.970** (2.395)	0.608** (2.001)
ROE	0.022*** (3.542)	0.019*** 3.256	0.009 (1.177)	0.004 (0.718)
DAR	-0.001 (-1.267)	-0.001 (-1.267)	0.003 (0.907)	0.001 (0.467)
RC	0.433*** (4.544)	0.279*** (2.898)	-0.019 (-0/056)	-0.321 (-1.289)
REV		0.328*** (3.762)		0.618*** (4.661)
Constant	-0.854* (-1.046)	-0.866 (-1.158)	-2.910** (-2.152)	-2.016* (-2.017)
adj R^2^	0.297	0.410	0.125	0.540

Note: *, **, *** indicate significant at the 10%, 5% and 1% levels, respectively.

## 5. Conclusions

### 5.1 Conclusions of the study

This study empirically analyzes the impact of directors’ information technology (IT) backgrounds and media coverage on the financing constraints of Internet startups using a sample from the New Third Board. The findings are as follows: firstly, directors’ IT backgrounds significantly reduce the financing constraints of Internet startups. Secondly, directors with IT backgrounds pay more attention to the emotional tone of media reports and use their professional knowledge to manage effective information output, which helps enterprises obtain favorable media reviews. Thirdly, positive media reports help reduce financing constraints and foster an investment and financing environment conducive to enterprise development. Moreover, these reports partially mediate the relationship between directors’ IT backgrounds and financing constraints. The results suggest that directors with IT backgrounds, due to their familiarity with information and networks, can help enterprises create a favorable media information environment. This environment enhances the market image desired by the enterprises and improves corporate financing efficiency [22].

### 5.2 Future prospects

Research on directors’ information technology (IT) backgrounds falls within the broader theme of entrepreneurial cognition [23]. Previous studies have predominantly focused on entrepreneurs’ intra- and extra-industry experiences, cognitive styles, and knowledge structures, without delving deeply into different types of experiences, styles, or knowledge [24]. Future research could explore the unique experiential context of an IT background to enhance the theoretical understanding of the cognitive mechanisms influencing entrepreneurs. Additionally, while directors’ IT backgrounds are generally assumed to positively impact corporate finance, the underlying mechanisms of this impact remain underexplored. Future studies could address this gap by introducing media reports as a mediating variable to better understand how IT backgrounds influence corporate finance.
